# Subgroups of Paediatric Acute Lymphoblastic Leukaemia Might Differ Significantly in Genetic Predisposition to Asparaginase Hypersensitivity

**DOI:** 10.1371/journal.pone.0140136

**Published:** 2015-10-12

**Authors:** Nóra Kutszegi, Ágnes F. Semsei, András Gézsi, Judit C. Sági, Viktória Nagy, Katalin Csordás, Zsuzsanna Jakab, Orsolya Lautner-Csorba, Krisztina Míta Gábor, Gábor T. Kovács, Dániel J. Erdélyi, Csaba Szalai

**Affiliations:** 1 Department of Genetics, Cell- and Immunobiology, Semmelweis University, Budapest, Hungary; 2 2nd Department of Paediatrics, Semmelweis University, Budapest, Hungary; 3 Department of Pediatrics and Pediatric Health Care Center, Faculty of Medicine, University of Szeged, Szeged, Hungary; 4 Central Laboratory, Heim Pal Children Hospital, Budapest, Hungary; University of Heidelberg, GERMANY

## Abstract

L-asparaginase (ASP) is a key element in the treatment of paediatric acute lymphoblastic leukaemia (ALL). However, hypersensitivity reactions (HSRs) to ASP are major challenges in paediatric patients. Our aim was to investigate genetic variants that may influence the risk to *Escherichia coli*-derived ASP hypersensitivity. Sample and clinical data collection was carried out from 576 paediatric ALL patients who were treated according to protocols from the Berlin—Frankfurt—Münster Study Group. A total of 20 single nucleotide polymorphisms (SNPs) in *GRIA1* and *GALNT10* genes were genotyped. Patients with *GRIA1* rs4958351 AA/AG genotype showed significantly reduced risk to ASP hypersensitivity compared to patients with GG genotype in the T-cell ALL subgroup (OR = 0.05 (0.01–0.26); p = 4.70E-04), while no such association was found in pre-B-cell ALL. In the medium risk group two SNPs of *GRIA1* (rs2055083 and rs707176) were associated significantly with the occurrence of ASP hypersensitivity (OR = 0.21 (0.09–0.53); p = 8.48E-04 and OR = 3.02 (1.36–6.73); p = 6.76E-03, respectively). Evaluating the genders separately, however, the association of rs707176 with ASP HSRs was confined only to females. Our results suggest that genetic variants of *GRIA1* might influence the risk to ASP hypersensitivity, but subgroups of patients can differ significantly in this respect.

## Introduction

Acute lymphoblastic leukaemia (ALL) is the most common paediatric malignancy, comprising approximately 25–30% of the annually registered cases of cancer among children [1,2]. Survival rates for paediatric and adolescent patients treated according to protocols by the Berlin—Frankfurt—Münster (BFM) Study Group have remarkably improved over the last decades with higher than 80% of children are cured [1,3,4]. The extensive use of L-asparaginase (ASP), which has been a crucial component of paediatric therapies since 1970s, contributes to this achievement [5–9].

ASP enzymes mainly derived from bacteria among which three different preparations from two bacteria are currently used: native and pegylated form of *Escherichia coli* ASP (*E*. *coli*-ASP and PEG-ASP, respectively) and an *Erwinia chrysanthemi*-derived ASP (*Erwinia*-ASP) with more or less different pharmacokinetic, pharmacodynamic and immunogenic properties [5]. Hypersensitivity reactions (HSRs) and silent inactivation via neutralizing antibody production against the enzyme are major challenges in paediatric patients, because both can lead to suboptimal treatment response. In addition, an anaphylactic reaction can be a potential threat to life requiring urgent interventions [7].

In BFM protocols *E*. *coli*-ASP is considered as first-line treatment. Clinical HSRs occur in up to 45% of paediatric ALL patients, which necessitate the discontinuation of *E*. *coli*-ASP administration and subsequent switch to PEG-ASP or *Erwinia*-ASP [10–12]. The most common manifestation of HSRs is urticaria [13]. However, the signs and symptoms can range from local reactions of erythema, swelling or pain at the injection site to severe symptoms, including laryngeal edema, bronchospasm, hypotension and occasionally systemic anaphylaxis [14]. Several risk factors of ASP hypersensitivity have been described including different preparations, dosage, route of administration, readministration after a hiatus and concomitant chemotherapy. However, this type of adverse reaction is unpredictable and exhibits large interindividual differences [11].

Recently, a genome wide association study has been carried out in an ethnically diverse population to identify germline genetic variations contributing to the risk of ASP allergy in children with ALL. In this study variants of the *GRIA1* (Glutamate Receptor, Ionotropic, AMPA 1) gene located at 5q33 have been found associated with ASP allergy [15]. This result has later been replicated in a small Slovenian population [16].

In gene association studies, due to the high number of inconclusive results, it is generally accepted that the role of a gene or a genetic variation can only be acknowledged, if it is confirmed by independent studies. In the present study our aim was to investigate the possible roles of genetic variations in the *GRIA1* gene in the susceptibility to *E*. *coli*-ASP hypersensitivity in a large Hungarian paediatric ALL population. Additionally, we also involved SNPs in *GALNT10* (Polypeptide N-Acetylgalactosaminyltransferase 10) gene, located also at 5q33, which in the original study were also found to be associated with ASP allergy in the discovery cohort, but could not be confirmed in a relatively small validation cohort.

## Patients and Methods

### Study population

Samples and clinical data collection were carried out from 576 paediatric acute lymphoblastic leukaemia (ALL) patients who were treated between 1990 and 2012 in 9 Hungarian paediatric haematology centres according to four consecutive chemotherapy protocols from the Berlin—Frankfurt—Münster Study Group (ALL-BFM 90, 95, ALL IC-BFM 2002 and 2009). These protocols in detail have been described elsewhere [3,4,17,18]. In this period of time two *E*. *coli*-ASP, Kidrolase (Jazz Pharmaceuticals, Inc.) or Asparaginase medac (Kyowa-Hakko) were available as first-line ASP preparations.

The treatment according to the different risk arms focusing on *E*. *coli*-ASP dosing schedules with the number of patients included are shown in S1 Table. Comparison of the treatments on different risk arms can also be found in S1 Text.

Data collection was carried out retrospectively from the files of the patients. We excluded 42 patients for the following reasons: lack of clinical information (n = 15), discontinuation of ASP administration not due to hypersensitivity (n = 1), switch to a different type of ASP not due to hypersensitivity (n = 1), genotyping call rate of the sample <50% (n = 28), significant deviation from the protocol (n = 6) and use of other treatment protocols (10). The inclusion criterion for controls was the completion of at least two blocks of *E*. *coli*-ASP containing treatment without any HSR. Therefore, we excluded further ten patients. Finally, a total of 505 patients were included in the analysis (Table 1).

**Table 1:** Patient characteristics.

**Gender (%)**	
Male	280 (55.4)
Female	225 (44.6)
**Years of age at diagnosis**	
Mean (± SD)	6.2 (±4.1)
Median (range)	4.9 (1–18)
**Risk category (%)**	
Low Risk, LR	147 (29.1)
Medium Risk, MR	305 (60.4)
High Risk, HR	53 (10.5)
**Immunophenotype (%)**	
pre-B ALL	402 (79.6)
T-ALL	69 (13.7)

The National Cancer Institute Common Toxicity Criteria (CTC) system v3.0 was used to assess the grade of hypersensitivity. For analyses, we regarded a case as asparaginase hypersensitivity when signs of allergic reactions or anaphylactic reactions CTC grade 1 and above were noted which occurred during the infusion or within 4 hours, plus the clinical team decided to discontinue, not readminister the given aparaginase preparation in the later treatments. The descriptions of the HSRs were included in the medical records of the patients.

Written informed consent was obtained from the study participants or from the next of kin, caretakers, or guardians on the behalf of the minors/children participants involved in the study. All the associated documents have been stored. The study was conducted according to the principles expressed in the Declaration of Helsinki and the whole study including the informed consent procedure were approved by the Hungarian Scientific and Research Ethics Committee of the Medical Research Council (ETT TUKEB; Case No.:8-374/2009-1018EKU 914/PI/08).

### Genes and polymorphisms

SNPs of *GRIA1* and *GALNT10* genes were searched for in online databases. The criteria of the SNP selection were the minor allele frequency ≥10% in Caucasian population, their estimated function based on dbSNP database of National Center of Biotechnology Information (NCBI; www.ncbi.nlm.nih.gov) or any relevant results published previously [19,20]. In case of *GALNT10* the whole 3’-UTR region were covered (S2 Table).

### Genotyping

In most cases, peripheral blood was obtained retrospectively from patients in remission phase. In some cases, only diagnostic peripheral blood or bone marrow cell suspension samples were available (n = 69). The genomic DNA was extracted by using the QIAamp DNA Blood Midi Kit (Qiagen, Valencia, CA, USA) according to the manufacturer’s instructions. In case of low amount or low concentration of DNA (a total of 87 samples) whole genome amplification was performed by using the REPLI-g Mini Kit (Qiagen, Valencia, CA, USA) according to the manufacturer’s instructions.

A total of 20 SNPs (10 in each gene) were genotyped by using KASPar-on-Demand prevalidated assays (LGC Genomics, Berlin, Germany) on 7900HT Fast Real-Time PCR System (Applied Biosystems, Foster City, CA, USA). The genotyping call rate for all SNPs was higher than 80%.

### Statistical analysis

Multi-adjusted logistic regression was performed by using IBM SPSS Statistic software, version 20.0 to test for associations. Gender, ALL immunophenotype, age at diagnosis, risk group, *E*. *coli*-ASP dosage during induction phase, BFM protocol and the polymorphisms in additive (11 vs. 12 vs. 22), dominant (11 vs. 12/22) and recessive (11/12 vs. 22) models were included in the analysis as categorical covariates (1, major allele; 2, minor allele). Odds ratios (ORs) and 95% confidence intervals (CIs) were obtained to estimate risks for each SNP to *E*. *coli*-ASP hypersensitivity. The analyses were performed not only for the overall ALL cohort, but also for subgroups created by gender, ALL immunophenotype (pre-B vs. T-ALL), age at diagnosis (≥10 years vs. <10 years) and risk category (standard vs. medium vs. high risk).

In order to deal with multiple comparisons the Benjamini-Hochberg false discovery rate (FDR) method with type I error rate of 5% (p≤ 6.76E-03) was applied as correction [21,22].

Power analysis was conducted by bootstrapping using R statistical software (R Foundation for Statistical Computing, Vienna, Austria; version 3.0.3) in the following way. First, we simulated 1 000 data replicates by sampling with replacement from the original dataset. Next, we applied logistic regression to each generated data set, and estimated power as the percentage of cases in which the null hypothesis was rejected.

Discrete-time survival analysis was used to assess the impact of SNPs on the minimum number of doses at which *E*. *coli*-ASP hypersensitivity developed. The hazard rate of the *i*-th patient was modeled by logistic regression:

$$logit\;h_{i}=\;\alpha D_{i}+\beta X_{i}$$

where ***D*** represents the minimum number of doses at which *E*. *coli*-ASP hypersensitivity developed by each study sample (more formally, for the *i*-th patient: ***D***_*ij*_ = 1, if the patient developed allergy *exactly* at the *j*-th dose of ASP, and ***D***_*ik*_ = 0 for all dose counts *k* where *k ≠ j*); *X*_*i*_ contains the value of the covariate (SNP) that might predict hazard function differences, and *α* and *β* are the appropriate regression coefficients. Analyses were performed using R statistical software.

The deviation from Hardy-Weinberg Equilibrium (HWE) was analysed by χ^2^ goodness-of-fit test using an online application (http://ihg.gsf.de/cgi-bin/hw/hwa1.pl).

## Results

We investigated the impact of *GRIA1* and *GALNT10* polymorphisms on the risk to *E*. *coli*-ASP hypersensitivity in paediatric ALL patients.

Out of the 20 SNPs, 18 were statistically evaluated, since two of them did not meet the requirements of HWE. The genotype and minor allele frequencies (MAF) with the results of the HWE are shown in S2 Table.

To examine the differences in genotype frequencies in patients with *E*. *coli*-ASP hypersensitivity and patients without HSRs to *E*. *coli*-ASP, multi-adjusted logistic regression analyses were applied on the overall cohort and on various subgroups.

Due to multiple testing, statistical corrections were applied. Applying FDR = 5% (p≤ 6.76E-03) significance threshold three SNPs in *GRIA1* gene showed statistically significant associations on certain subgroups.

The association of rs4958351 with *E*. *coli*-ASP hypersensitivity was not replicated in our total study cohort. The allele and genotype distributions for each SNP can be found in S3 Table. When we evaluated the associations between SNPs and *E*. *coli*-ASP hypersensitivity in T-cell and pre-B-cell ALL separately, in case of rs4958351 a significant difference between the subgroups was detected. In the dominant model patients with rs4958351 AA/AG genotype showed significantly reduced risk to ASP hypersensitivity compared to patients with GG genotype in the T-ALL subgroup (OR = 0.05 (0.01–0.26); p = 4.70E-04; power = 0.78); Table 2). To examine a more homogeneous group we also analysed T-ALL patients on MR arm. In this way, the association still could be detected (N = 50; OR = 0.05 (0.01–0.29); p = 8.43E-04).

**Table 2:** Associations of rs4958351 with the occurrence of *E*. *coli*-ASP hypersensitivity in the total cohort and in the immunophenotypic subgroups.

		rs4958351		
		GG vs. AG/AA		
Subgroup		N	p value	OR (95% CI)
**Total**		464	0.500	0.87 (0.58–1.31)
**Immunophenotype**	**pre-B ALL**	364	0.522	1.16 (0.73–1.85)
	**T-ALL**	**66**	**4.70E-04**	**0.05 (0.01–0.26)**

Results that reached the significance threshold (FDR(α) = 5%; p≤ 6.76E-03) are in bold.

The overall rate of *E*. *coli*-ASP hypersensitivity was 37% (37%, 30% and 77% in standard (SR), medium (MR) and high risk (HR) group, respectively). We found association between two polymorphisms (rs2055083 and rs707176) and the occurrence of ASP hypersensitivity in the MR group. On the one hand, in the dominant model, patients with rs2055083 AA/AG genotype had significantly lower risk to develop ASP hypersensitivity compared to patients with GG genotype (OR = 0.21 (0.09–0.53); p = 8.48E-04; power = 0.97; Table 3).

**Table 3:** Associations of rs2055083 with the occurrence of *E*. *coli*-ASP hypersensitivity in the total cohort and in different risk groups.

		rs2055083		
		GG vs. AG/AA		
Subgroup		N	p value	OR (95% CI)
**Total**		490	0.104	0.66 (0.39–1.09)
**Risk category**	**SR**	141	0.090	2.08 (0.89–4.84)
	**MR**	**298**	**8.48E-04**	**0.21 (0.09–0.53)**
	**HR**	51	0.245	4.73 (0.34–64.90)

Results that reached the significance threshold (FDR(α) = 5%; p≤ 6.76E-03) are in bold.

On the other hand, in recessive model, patients with CC genotype of rs707176 had significantly higher risk to have ASP hypersensitivity until the end of the reinduction compared to patients harbouring at least one T allele (OR = 3.02 (1.36–6.73); p = 6.76E-03; power = 0.73; Table 4). The risk to *E*. *coli*-ASP hypersensitivity was approximately four times higher for female patients with CC genotype compared to patients with at least one T allele (OR = 4.03 (1.48–10.94), p = 6.28E-03; power = 0.78), while no such association was found in male patients.

**Table 4:** Associations of rs707176 with the occurrence of E. coli-ASP hypersensitivity in the total cohort and in different subgroups.

		rs707176^a^		
		CT/TT vs. CC		
Subgroup		N	p value	OR (95% CI)
**Total**		477	0.041	1.90 (1.02–3.48)
**Risk category**	**SR**	136	0.689	1.25 (0.42–3.68)
	**MR**	**292**	**6.76E-03**	**3.02 (1.36–6.73)**
	**HR**	49	0.385	0.36 (0.03–3.66)
**Gender**	**Male**	263	0.770	1.14 (0.48–2.66)
	**Female**	**214**	**6.28E-03**	**4.03 (1.48–10.94)**

Results that reached the significance threshold (FDR(α) = 5%; p≤ 6.76E-03) are in bold.

^a^ the cofactor of ASP dosage during induction was not included in the analysis due to numerical problems of the logistic regression model created by the presence of cell values equal to zero.

When we evaluated the genders separately in each risk category, it turned out that in the MR group the association of rs707176 with ASP HSR was confined only to females with a very high OR (OR = 11.56 (2.56–52.27); p = 1.48E-03; Table 5).

**Table 5:** Associations of rs707176 with the occurrence of E. coli-ASP hypersensitivity in subgroups created by risk category and gender.

			rs707176^a^		
			CT/TT vs. CC		
Subgroup			N	p value	OR (95% CI)
**Risk category**	**SR**	**Male**	65	0.806	1.24 (0.23–6.77)
		**Female**	71	0.726	1.30 (0.30–5.74)
	**MR**	**Male**	165	0.767	1.19 (0.38–3.77)
		**Female**	**127**	**1.48E-03**	**11.56 (2.56–52.27)**
	**HR**	**Male**	n.a.^b^	n.a.^b^	n.a.^b^
		**Female**	n.a.^b^	n.a.^b^	n.a.^b^

Results that reached the significance threshold (FDR(α) = 5%; p≤ 6.76E-03) are in bold.

^a^ the cofactor of ASP dosage during induction was not included in the analysis due to numerical problems of the logistic regression model created by the presence of cell values equal to zero.

^b^ not analysed due to the presence of cell values equal to zero.

**Fig 1:**
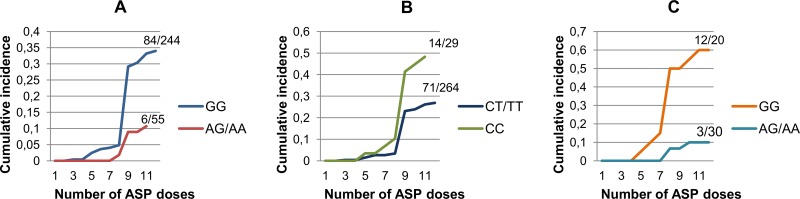
The cumulative incidence of *E*. *coli*-ASP hypersensitivity with the number of ASP doses. (A) In case of rs2055083 the cumulative incidence of *E*. *coli*-ASP by the end of the of the second block on MR arm was 34% (84 out of 244) and 11% (6 out of 55) for patients with GG and AA/AG genotypes, respectively. (B) In case of rs707176 the same value was 48% (14 out of 29) and 27% (71 out of 264) for patients with CC and CT/TT genotypes, respectively. (C) Analysing T-ALL patients on MR arm the cumulative incidence of *E*. *coli*-ASP hypersensitivity by the end of the of the second block was 60% (12 out of 20) and 10% (3 out of 30) for patients with GG and AA/AG genotypes, respectively.

The highest incidence of HSRs to *E*. *coli*-ASP in the MR group was observed during the first dose of ASP in the reinduction (approximately 70% of the overall numbers of cases) after a three-month-long break in ASP therapy. By the end of the *E*. *coli*-ASP treatment the cumulative incidence of *E*. *coli*-ASP hypersensitivity was 34% (84 out of 244) and 11% (6 out of 55) for patients with rs2055083 GG and AA/AG genotypes (p = 1.3E-3), respectively (Fig 1A). In case of rs707176 this value was 48% (14 out of 29) and 27% (71 out of 264) for patients with CC and CT/TT genotypes (p = 3.1E-2), respectively (Fig 1B). In order to be comparable we analysed T-ALL subgroup restricted to MR patients: the cumulative incidence of *E*. *coli*-ASP hypersensitivity was 60% (12 out of 20) and 10% (3 out of 30) for patients with GG and AA/AG genotypes (p = 5.0E-4), respectively (Fig 1C). After completing the reinduction therapy on MR arm (a total of 12 doses of *E*. *coli*-ASP) without any HSR to *E*. *coli*-ASP none of the patients developed *E*. *coli*-ASP hypersensitivity in further blocks.

Furthermore, focusing on MR patients we used discrete-time survival analysis to assess the effect of the SNPs on the minimum number of doses at which *E*. *coli*-ASP hypersensitivity developed. A strong effect of rs2055083 could be detected on the minimum number of doses. The hazard ratio of the minor A allele in dominant model was 0.25 (N = 299; 95% CI: (0.11–0.60); p = 1.8E-03). This means that the hazard rate of patients who have at least one A allele is approximately 75% less than that of the patients who are homozygous to the G allele. In contrast, the minor C allele of rs707176 in recessive model was proved to be a strong risk factor (N = 293; hazard ratio: 2.38; 95% CI: (1.23–4.62); p = 0.0105). We also found a strong effect of rs4958351 on the minimum number of doses in the T-ALL subgroup on MR arm. The hazard rate of patients harbouring at least one A allele was about 91% less than patients with GG genotype (N = 50; hazard ratio: 0.09; 95% CI: (0.02–0.36); p = 6.1E-04). Hence, the A allele of rs4958351 appeared to be a preventive factor in T-cell ALL.

Univariate and multivariate logistic regression was applied to evaluate the differences in the rate of *E*. *coli*-ASP hypersensitivity in various subgroups. Patients on HR treatment arm had significantly increased odds of developing *E*. *coli*-ASP hypersensitivity compared to those on SR and MR arm (OR = 5.88 (2.85–12.15); p = 2.00E-06 and OR = 10.50 (2.82–39.02); p = 4.49E-04, respectively). We found no differences in *E*. *coli*-ASP hypersensitivity rate between further subgroups (Table 6).

**Table 6:** Differences between subgroups in *E*. *coli*-ASP hypersensitivity.

Subgroup		N cases / N total (%)	Univariate p value	OR (95% CI)	Multivariate p value	OR (95% CI)
**Gender**	Male	99/280 (35.4)	ref.*		ref.*	
	Female	87/225 (38.7)	0.444	1.15 (0.80–1.66)	0.225	1.27 (0.86–1.87)
**Immunophenotype**	pre-B ALL	152/402 (37.8)	ref.*		ref.*	
	T-ALL	26/69 (37.7)	0.984	0.99 (0.59–1.68)	0.854	0.99 (0.47–1.56)
**Age at diagnosis**	<10 years	151/413 (36.6)	ref.*		ref.*	
	≥10 years	35/92 (38.0)	0.790	1.07 (0.67–1.70)	0.921	1.03 (0.60–1.75)
**Risk Category**	Standard Risk	54/147 (36.7)	ref.*		ref.*	
	Medium Risk	91/305 (29.8)	0.142	0.73 (0.48–1.11)	0.512	0.86 (0.54–1.37)
	High Risk	41/53 (77.4)	**2.00E-06**	**5.88 (2.85–12.15)**	**4.49E-04**	**10.50 (2.82–39.02)**
Dosage of E. coli-ASP during induction**	5000 IU/m2/d	53/138 (38.4)	ref.*		ref.*	
	10000 IU/m2/d	91/310 (29.4)	0.059	0.67 (0.44–1.02)	0.398	1.93 (0.42–8.78)

Results that reached the significance threshold (p≤ 0.05 in univariate and FDR(α) = 5%; p≤ 6.76E-03 in multivariate analysis) are in bold.

* ref. refers to the reference group to which the others groups are to be compared

** on standard and medium risk arm

## Discussion

The aim of our study was to investigate the impact of genetic variants of *GRIA1* and *GALNT10* genes on ASP allergy in a large Hungarian population of 576 ALL patients. We identified one synonymous and two intronic *GRIA1* SNPs associated with *E*. *coli*-ASP hypersensitivity on certain ALL subgroups. We could find no association regarding the *GALNT10* polymorphisms.

Previously, Chen *et al*. analysed more than 500,000 SNPs in a genome-wide association study of 485 children with ALL. They found that the rs4958351 and four additional intronic SNPs of the *GRIA1* gene on chromosome 5q33 region were associated with ASP hypersensitivity [15]. Recently, these results were replicated in an independent, relatively small Slovenian population of 146 paediatric ALL patients [16]. Chen *et al*. also identified genetic variants of *GALNT10* (located in the same region) in the initial cohort of 322 paediatric patients, but these associations could not be detected in the smaller validation cohort of 163 children [15].

*GRIA1* encodes a subunit (GluR1) of the ionotropic alpha-amino-3-hydroxy-5-methyl-4-isoxazole propionate (AMPA) receptor. These receptors are homomeric or heteromeric protein complexes consisting of GluR1-4 subunits, which are arranged to form a ligand-gated ion channel transmitting glutamatergic signals in the central nervous system (http://www.genecards.org/cgi-bin/carddisp.pl?gene=GRIA1).

Recent studies have shown that glutamate acts also as an immunomodulator in addition to being a neurotransmitter [15,16,23]. For the first time, in 2001 Lombardi *et al*. described the presence of ionotropic glutamate receptors (iGluRs) on the surface of human T-lymphocytes. The same study has revealed the functionality of AMPA receptors by potentiation of Ca^2+^ influx upon T-cell activation [24]. Later, other studies have demonstrated that glutamate induced both a pro-adhesive and a pro-migratory effect on naïve/resting T-cells by acting via its AMPA receptors [25–27]. Moreover, the expression of several glutamate receptors and transporters has been described in various types of immune cells including dendritic cells (DCs). DCs release glutamate upon interacting with T-lymphocytes in lymph nodes. Pacheco *et al*. demonstrated that during this interaction glutamate acts as a highly effective regulator in the initiation of T-cell mediated immune responses [27]. Besides, in addition to the *ab ovo* elevated level of plasma glutamate due to malignancy, the glutaminase activity of *E*. *coli*-ASP results in a significant rise of the cleavage product glutamate during treatment with a high interindividual variability [28]. Taken together, these data suggest that genetic polymorphisms of glutamate signalling pathways in immune system may influence the risk of developing ASP hypersensitivity.

The rs4958351 SNP showed no association with *E*. *coli*-ASP hypersensitivity in our total study cohort. However, we found that the effect of the A allele drastically differs on the susceptibility to ASP hypersensitivity in the different subtypes of ALL. While it is associated with significantly reduced risk to HSR in T-cell ALL (OR = 0.05), it is associated with a slightly although not significantly increased risk to HSR in pre-B-cell ALL. In contrast, Chen *et al*. and Rajić *et al*. have identified the A allele as a risk allele in the whole ALL cohort. However, perhaps because of the low number of T-ALL patients in their study cohorts, the different immunophenotypic subgroups have not been evaluated separately. To our knowledge, ours is the first study on the relation of rs4958351 to the risk of *E*. *coli*-ASP hypersensitivity in T-ALL as a separate subgroup; hence this opposite association in this subgroup of ALL patients could have been hidden up to this point.

In our study the intronic rs2055083 polymorphism in the *GRIA1* gene appeared to be a strong preventive factor of *E*. *coli*-ASP hypersensitivity in a homogeneous cohort of MR ALL patients. No result has been published so far related to rs2055083. According to the results of the 1000 Genomes Project the rs2055083 is in linkage disequilibrium (LD) with another intronic SNP rs10515693 (http://www.ensembl.org/Homo_sapiens/Variation/HighLD?db=core;r=5:153487464-153667465;v=rs2055083;vdb=variation;vf=1720088#13083_tablePanel; r^2^ = 0.953; D’ = 1.000; Ensembl release 81—July 2015) in the CEPH population. However, in the European (EUR) super population the regarding values were lower (r^2^ = 0.7; D’ = 0.85). Using the online Variant Effect Predictor (VEP) tool of Ensembl it has been revealed that the latter polymorphism is located in a regulatory region (ENSR00001294367), which is active in a human B-lymphocyte cell line GM12878 and acts as an enhancer (http://www.ensembl.org/Homo_sapiens/Regulation/Summary?db=core;fdb=funcgen;r=5:153554305-153557304;rf=ENSR00001294367;tl=gEE3ksgDRibSzsCu-737322). Based on this, one possible reason of association between rs2055083 and ASP allergy that rs2055083 is in LD with a regulatory variant in B-lymphocytes.

It has been shown in an Italian case-control association study that the allele distributions of rs707176 were different in DSM-IV-TR (Diagnostic and Statistical Manual of Mental Disorders, Fourth Edition, Text Revision) schizophrenia controls and in cases [20]. In our study population this SNP, which is a synonymous C/T transition in exon 3 of the *GRIA1* gene, was associated with *E*. *coli*-ASP hypersensitivity in MR subgroup (p = 6.76E-03). The risk of CC homozygotes to HSR was very high in females (OR = 4.03 (1.48–10.94)), while no such effect could be observed in males which suggests a sex difference in the genetic background of ASP hypersensitivity.

All the genetic variations we genotyped in *GRIA1* gene were intronic or synonymous variants. The functionality of these so-called silent polymorphisms is not yet clear. Intronic polymorphisms in the glutamate receptor subunit *GluR2* have roles in directing RNA editing of the *GluR2* coding sequence [15,16,29]. Furthermore, it is known that *GRIA1* has alternatively spliced transcript variants encoding different isoforms (http://www.genecards.org/cgi-bin/carddisp.pl?gene=GRIA1). Emerging data show that the fine balance of alternative splicing isoforms may also be altered by variants of exonic or intronic splicing regulatory elements [30]. These findings suggest mechanisms by which these silent polymorphisms could influence gene function.

We tested the differences in the occurrence of *E*. *coli*-ASP hypersensitivity by logistic regression between subgroups created by gender, age at diagnosis, risk category, immunophenotype of leukaemia and the dose of *E*. *coli*-ASP during induction on SR and MR arms. HR patients were at approximately six times higher odds of developing *E*. *coli*-ASP hypersensitivity compared to SR patients (p = 2.00E-06). The occurrence of *E*. *coli*-ASP hypersensitivity did not differ significantly between the SR and the MR groups. These findings can be explained by the considerably different treatment with more and greater doses of ASP as well as with many breaks in ASP therapy on HR arm compared to SR and MR arms.

The highest occurrence of *E*. *coli*-ASP hypersensitivity reactions was after a three-month-long break (consolidation phase) in ASP treatment during the first dose of reinduction on MR arm. It is in accordance with previously observed phenomena that reexposure of ASP after a hiatus increases the risk to HSRs [10,11]. The proliferation and antibody production of competent B-cells during consolidation can be one of the possible explanations [16,31].

This study has several strengths and limitations. First, although the rate of relapsed patients of our cohort is similar to the relapse rate of the whole ALL population, the rate of died patients, however, is lower in our study population. Patients who died during the chemotherapy due to therapy resistant progressive disease or due to infections or toxicities of therapy are underrepresented in our ALL cohort. Furthermore, HSR data to *E*.*coli*-ASP was collected retrospectively from the files of the patients. This manner does not allow meticulous documentation and fine grading of hypersensitivity reactions.

Our findings pertain to reactions to *E*. *coli*-ASP. Fernandez et al. recently reported that the association between the rs4958351 variant and hypersensitivity was strongest among patients receiving native *E*. *coli* asparaginase compared to PEG-asparaginase [32].

A small proportion of our DNA samples had been originated from the diagnostic clone of lymphoblasts. We hypothesized that the probability of the genetic alteration of the investigated polymorphisms was extremely low during leukaemogenesis. To verify this, we also performed the analysis excluding these samples and calculated the allele frequencies for the remission and for the diagnostic samples. Even in this way, the results remained statistically significant and we could not find substantial differences in the allele frequencies either (data not shown).

In conclusion, in our relatively large population we confirmed that genetic variations in the *GRIA1* gene could significantly influence the risk of ASP hypersensitivity. Furthermore, we found that the direction of the effect could be significantly different in the different subgroups of patients. In addition to previously published associations, we identified novel polymorphisms, which may serve to enlighten new details in the genetic background of *E*. *coli*-ASP hypersensitivity reactions. Replication in other cohorts is warranted to confirm our findings. Functional analysis of variants is needed, especially in case of the rs4958351 in T-ALL to elucidate its role in ASP allergy development.

## Supporting Information

S1 Table The treatment focusing on ASP dosing schedules according to different risk arms. (PDF)

S2 Table Genes and SNPs included in the analysis. (PDF)

S3 Table Allele and genotype distributions of the total cohort of ALL patients. (PDF)

S1 Text Comparison of the different chemotherapy protocols. (PDF)
