# Supplementary material for: Subgroups of Paediatric Acute Lymphoblastic Leukaemia Might Differ Significantly in Genetic Predisposition to Asparaginase Hypersensitivity
Source: PLoS One. 2015 Oct 12;10(10):e0140136. doi: 10.1371/journal.pone.0140136 (PMC4601692; doi:10.1371/journal.pone.0140136)
Supplement: S1 Table — (PDF) [file pone.0140136.s001.pdf]

Supplementary Table 1. The treatment focusing on ASP dosing schedules according to (A) SR, (B) MR and (C) HR arm with the number of the patients included.

A

Time			BFM protocol/treatment arm			
			ALL-BFM 90	ALL-BFM 95	ALL IC-BFM 2002	
			SR	SR	SR standard	SR experimental
	N (%)		9 (6)	85 (59)	43 (30)	7 (5)
	**Induction**	Block	Protocol I/1 (33 d)			
		E. coli-ASP dosage (IU/m^2/d)	10 000 p.i.* (1 h) x 8 q 3 days		5000 p.i.* (1 h) x 8 q 3 days	
			**Intensification (28 d)<br>Consolidation (56 d)**			
	**Reinduction**	Block	Protocol II/1 (28 d)			Protocol III/1 (14 d)
		E. coli-ASP dosage (IU/m^2/d)	10 000 p.i.* (1 h) x 4 on day: 8, 11, 15, 18			10 000 p.i.* (1 h) x 4 on day: 1, 4, 8, 11
			**Reintensification (14 d)<br>Maintenance**			**Reintensification (14 d)<br>Interim maintenance (10 weeks)**
	**Late-reinduction**	Block	-			Protocol III/1 (14 d)
		E. coli-ASP dosage (IU/m^2/d)				10 000 p.i.* (1 h) x 4 on day: 1, 4, 8, 11

B

Time			BFM protocol/treatment arm				
			ALL-BFM 90	ALL-BFM 95	ALL IC-BFM 2009	ALL IC-BFM 2002	
			MR standard	MR	MR standard	MR standard	MR experimental
	N (%)		90 (30)	126 (41)	4 (1)	61 (20)	23 (8)
	**Induction**	Block	Protocol I/1 (33 d)				
		E. coli-ASP dosage (IU/m^2/d)	10 000 p.i.* (1 h) x 8 q 3 days		5000 p.i.* (1 h) x 8 q 3 days		
	**Intensification (28 d) Consolidation (56 d)**						
	**Reinduction**	Block	Protocol II/1 (28 d)				Protocol III/1 (14 d)
		E. coli-ASP dosage (IU/m^2/d)	10 000 p.i.* (1 h) x 4 on day: 8, 11, 15, 18				10 000 p.i.* (1 h) x 4 on day: 1, 4, 8, 11
			**Reintensification (14 d) Maintenance**				**Reintensification (14 d) Interim maintenance (28 d)**
	**Late-reinduction**	Block	-				Protocol III/1 (14 d)
		E. coli-ASP dosage (IU/m^2/d)					10 000 p.i.* (1 h) x 4 on day: 1, 4, 8, 11
							**Reintensification (14 d) Interim maintenance (28 d)**
	**Late-reinduction**	Block	-				Protocol III/1 (14 d)
		E. coli-ASP dosage (IU/m^2/d)					10 000 p.i.* (1 h) x 4 on day: 1, 4, 8, 11

C

Time			ALL-BFM 90	ALL-BFM 95	ALL IC-BFM 2009	ALL IC-BFM 2002	
			HR	HR	HR standard	HR standard	HR experimental
N (%)			9 (17)	21 (40)	1 (2)	16 (30)	6 (11)
BFM protocol/treatment arm							
**Induction**	Block		Protocol IA (29 d)		Protocol I/1 (33 d)		
	E. coli-ASP dosage (IU/m^2/d)		10 000 p.i.* (1 h) x 6 q 3 days		5000 p.i.* (1 h) x 8 q 3 days		
**Intensified consolidation**			-		**Intensification (28 d)**		
	Block		(HR'-1/HR'-2/HR'-3)x3 (5x3x3 d)	(HR'-1/HR'-2/HR'-3)x2 (5x3x2 d)			(HR'-1/HR'-2/HR'-3)x1 (5x3 d)
	E. coli-ASP dosage (IU/m^2/d)		25 000 p.i.* (6 h) on day 6 in each element		25 000 p.i.* (2 h) x 2 on day 6 and 11 in each element		
**Reinduction**	Block		**-**	Protocol II/1 (28 d)			Protocol III/1 (14 d)
	E. coli-ASP dosage (IU/m^2/d)		-	10 000 p.i.* (1 h) x 4 on day: 8, 11, 15, 18			10 000 p.i.* (1 h) x 4 on day: 1, 4, 8, 11
			**Maintenance**	**Reintensification (14 d)<br>Maintenance**			**Reintensification (14 d)<br>Interim maintenance (28 d)**
**Late-reinduction**	Block		-				Protocol III/1 (14 d)
	E. coli-ASP dosage (IU/m^2/d)		-				10 000 p.i.* (1 h) x 4 on day: 1, 4, 8, 11

			**Reintensification (14 d)<br>Interim maintenance<br>(28 d)**
**Late-reinduction**	Block	-	Protocol III/1 (14 d)
	E. coli-ASP dosage (IU/m^2/d)		10 000 p.i.* (1 h) x 4 on day: 1, 4, 8, 11

In case of four patients the treatment protocol data was not available. These patients were not included in the multi-adjusted analyses hence they are not shown in these tables.

*p.i. refers to *per infusionem*
