# Supplementary material for: Subgroups of Paediatric Acute Lymphoblastic Leukaemia Might Differ Significantly in Genetic Predisposition to Asparaginase Hypersensitivity
Source: PLoS One. 2015 Oct 12;10(10):e0140136. doi: 10.1371/journal.pone.0140136 (PMC4601692; doi:10.1371/journal.pone.0140136)
Supplement: S2 Table — (PDF) [file pone.0140136.s002.pdf]

Supplementary Table 2. Genes and SNPs included in the analysis

Gene	SNP ID	Alleles (1/2)[a]	Position [b]	Function [c]	MAF[d]	Genotype Frequency			HWE[e] (p value)	SNP Call Rate (%)
						1/1	1/2	2/2		
GRIA1	rs548294	C/T	chr5:153488877	2KB upstream	0.36	41.7	44.4	13.8	0.42	94
	rs2055083	G/A	chr5:153577458	intron	0.10	80.2	19.0	0.8	0.54	96
	rs1994862	C/G	chr5:153609350	intron	0.32	46.2	43.8	10.0	0.87	83
	rs707176	T/C	chr5:153650400	synonymous	0.33	45.3	43.2	11.4	0.62	93
	rs970078	G/T	chr5:153657654	intron	0.48	27.5	48.1	24.4	0.40	96
	rs11167640	T/C	chr5:153752746	intron	0.21	64.1	30.6	5.2	0.17	96
	rs11749754	G/A	chr5:153767331	intron	0.18	68.2	28.3	3.6	0.53	87
	rs4958351	G/A	chr5:153790814	intron	0.34	42.1	47.2	10.7	0.31	90
	rs1461224	T/G	chr5:153806677	intron	0.48	29.6	43.9	26.5	**0.01**	83
	rs2926833	G/A	chr5:153814103	500B downstream	0.17	69.5	27.6	2.8	0.95	90
GALNT10	rs17552639	A/G	chr5:154271899	intron	0.19	65.3	31.8	2.9	0.33	95
	rs11167667	C/T	chr5:154274385	intron	0.42	34.9	47.1	18.0	0.52	95
	rs2443526	T/C	chr5:154276970	intron	0.45	33.6	43.4	23.0	**0.007**	95
	rs6580076	C/T	chr5:154404193	synonymous	0.16	70.5	27.9	1.7	0.20	93
	rs888979	A/G	chr5:154417315	3'-UTR	0.31	48.5	40.4	11.0	0.19	93
	rs7710430	C/T	chr5:154417717	3'-UTR	0.31	48.6	40.7	10.6	0.29	93
	rs3172941	C/T	chr5:154418320	3'-UTR	0.30	50.2	39.2	10.6	0.15	83
	rs10796	G/C	chr5:154418808	3'-UTR	0.20	64.9	30.1	5.0	0.17	93
	rs2277937	T/C	chr5:154419605	3'-UTR	0.29	50.5	40.4	9.1	0.58	96
	rs7244	G/A	chr5:154420953	3'-UTR	0.18	68.8	27.4	3.8	0.26	91

P values (Pearson's goodness-of-fit chi-square (degree of freedom = 1)) under the significance threshold (p≤0.05) are in bold.

[a] alleles on the forward strand; 1, major allele; 2, minor allele

[b] position according to GRCh38 Annotation Release 106

[c] estimated function according to dbSNP 142

[d] minor allele frequency

[e] Hardy-Weinberg equilibrium
