# Supplementary material for: Subgroups of Paediatric Acute Lymphoblastic Leukaemia Might Differ Significantly in Genetic Predisposition to Asparaginase Hypersensitivity
Source: PLoS One. 2015 Oct 12;10(10):e0140136. doi: 10.1371/journal.pone.0140136 (PMC4601692; doi:10.1371/journal.pone.0140136)
Supplement: S3 Table — (PDF) [file pone.0140136.s003.pdf]

Supplementary Table 3. Allele and genotype distributions of ALL patients

Gene	SNP	Genotype	N	Category	N	MAF[a]
GRIA1	rs548294	CC	124	controls	309	0.37
		CT	143			
		TT	42			
		CC	78	cases	175	0.35
		CT	72			
		TT	25			
	rs2055083	GG	245	controls	313	0.11
		AG	65			
		AA	3			
		GG	151	cases	181	0.09
		AG	29			
		AA	1			
	rs1994862	CC	127	controls	280	0.32
		CG	126			
		GG	27			
		CC	71	cases	149	0.32
		CG	62			
		GG	16			
	rs707176	TT	141	controls	304	0.32
		CT	134			
		CC	29			
		TT	77	cases	177	0.36
		CT	74			
		CC	26			
	rs970078	GG	85	controls	312	0.48
		GT	152			
		TT	75			
		GG	51	cases	183	0.49
		GT	86			
		TT	46			
	rs11167640	TT	208	controls	315	0.20
		CT	89			
		CC	18			
		TT	110	cases	181	0.22
		CT	63			
		CC	8			

	rs11749754	GG	197	controls	291	0.18
		AG	86			
		AA	8			
		GG	109	cases	158	0.18
		AG	41			
		AA	8			
	rs4958351	GG	124	controls	300	0.35
		AG	144			
		AA	32			
		GG	72	cases	166	0.34
		AG	76			
		AA	18			
	rs1461224	TT	84	controls	276	0.49
		GT	115			
		GG	77			
		TT	42	cases	150	0.48
		GT	72			
		GG	36			
	rs2926833	GG	211	controls	298	0.16
		AG	77			
		AA	10			
		GG	111	cases	165	0.17
		AG	51			
		AA	3			
GALNT10	rs17552639	AA	201	controls	310	0.19
		AG	100			
		GG	9			
		AA	119	cases	180	0.18
		AG	56			
		GG	5			
	rs11167667	CC	115	controls	309	0.40
		CT	141			
		TT	53			
		CC	56	cases	181	0.44
		CT	90			
		TT	35			
	rs2443526	TT	110	controls	310	0.44
		CT	130			
		CC	70			
		TT	54	cases	178	0.47
		CT	82			
		CC	42			

rs6580076	CC	215	controls	304	0.16
	CT	83			
	TT	6			
	CC	124	cases	177	0.16
	CT	51			
	TT	2			
rs888979	AA	146	controls	306	0.32
	AG	125			
	GG	35			
	AA	87	cases	174	0.30
	AG	69			
	GG	18			
rs7710430	CC	146	controls	307	0.32
	CT	125			
	TT	36			
	CC	87	cases	172	0.29
	CT	70			
	TT	15			
rs3172941	CC	139	controls	277	0.30
	CT	108			
	TT	30			
	CC	75	cases	149	0.30
	CT	59			
	TT	15			
rs10796	GG	202	controls	309	0.19
	CG	94			
	CC	13			
	GG	109	cases	170	0.21
	CG	50			
	CC	11			
rs2277937	TT	161	controls	314	0.29
	CT	125			
	CC	28			
	TT	89	cases	181	0.30
	CT	75			
	CC	17			
rs7244	GG	207	controls	298	0.17
	AG	80			
	AA	11			
	GG	117	cases	173	0.18
	AG	49			
	AA	7			

[a] Minor Allele Frequency
