# Supplementary material for: Subgroups of Paediatric Acute Lymphoblastic Leukaemia Might Differ Significantly in Genetic Predisposition to Asparaginase Hypersensitivity
Source: PLoS One. 2015 Oct 12;10(10):e0140136. doi: 10.1371/journal.pone.0140136 (PMC4601692; doi:10.1371/journal.pone.0140136)
Supplement: S1 Text — (PDF) [file pone.0140136.s004.pdf]

S1 Text. Comparison of the different chemotherapy protocols

In aspect of ASP administration patients received virtually the same treatment on the standard and medium risk arm. The concomitant chemotherapeutic drugs were prednisone *p.o./i.v.*, vincristine *i.v.*, daunorubicin *p.i.* and methotrexate *i.th*. in induction followed by dexamethason *p.o./i.v.*, vincristine *i.v.*, doxorubicin *i.v.* and methotrexate *i.th.* (for CNS positive patients) in reinduction and late-reinduction.

On the high risk arm after an identical induction phase patients received ASP containing blocks as consolidation therapy (HR-1'/HR-2'/HR-3') for one, two or three times depending on BFM protocol. In consolidation 25 000 IU/m2/d *E. coli*-ASP was administered once or twice in each 5-11-day-long block along with the following agents: dexamethason *p.o./i.v.*, vincristine *i.v.*, methotrexate *p.i.*, cyclophosphamide *p.i.*, cytarabine *p.i.* and methotrexate/cytarabine/prednisolone *i.th.* in HR-1'; dexamethason *p.o./i.v.*, vindesine *i.v.*, methotrexate *p.i.*, ifosfamide *p.i.*, daunorubicin *p.i.* and methotrexate/cytarabine/prednisolone *i.th.* in HR-2' and dexamethason *p.o./i.v.*, cytarabine *p.i.*, etoposide *p.i.* and methotrexate/cytarabine/prednisolone *i.th.* in HR-3'. In ALL-BFM 90 protocol three HR-1'/HR-2'/HR-3' blocks were followed by maintenance therapy, whereas in other protocols patients received reinduction and/or late-reinduction therapy (same as described above) after one or two series of HR-1'/HR-2'/HR-3' blocks (1-4).

p.o. refers to *per os*

i.v. refers to intravenous

i.th. refers to intrathecal

p.i. refers to *per infusionem*

1. Erdelyi DJ, Kamory E, Csokay B, Andrikovics H, Tordai A, Kiss C, et al. Synergistic interaction of ABCB1 and ABCG2 polymorphisms predicts the prevalence of toxic encephalopathy during anticancer chemotherapy. Pharmacogenomics J. 2008 Oct;8(5):321-7. PubMed PMID: 17938643. Epub 2007/10/17. eng.
2. Moricke A, Reiter A, Zimmermann M, Gadner H, Stanulla M, Dordelmann M, et al. Risk-adjusted therapy of acute lymphoblastic leukemia can decrease treatment burden and improve survival: treatment results of 2169 unselected pediatric and adolescent patients enrolled in the trial ALL-BFM 95 (vol 111, pg 4477, 2008). Blood. 2009 Apr 30;113(18):4478-. PubMed PMID: WOS:000265846300046.
3. Stary J, Zimmermann M, Campbell M, Castillo L, Dibar E, Donska S, et al. Intensive Chemotherapy for Childhood Acute Lymphoblastic Leukemia: Results of the Randomized Intercontinental Trial ALL IC-BFM 2002. Journal of Clinical Oncology. 2014 Jan 20;32(3):174-+. PubMed PMID: WOS:000330627900004.
4. Gezsi A, Lautner-Csorba O, Erdelyi DJ, Hullam G, Antal P, Semsei AF, et al. In interaction with gender a common CYP3A4 polymorphism may influence the survival rate of chemotherapy for childhood acute lymphoblastic leukemia. Pharmacogenomics J. 2014 09/30/online.
